# Supplementary material for: Structural basis of frizzled 7 activation and allosteric regulation
Source: Nat Commun. 2024 Aug 28;15:7422. doi: 10.1038/s41467-024-51664-4 (PMC11358414; doi:10.1038/s41467-024-51664-4)
Supplement: Supplementary file 1 — Supplementary Information [file 41467_2024_51664_MOESM1_ESM.pdf]

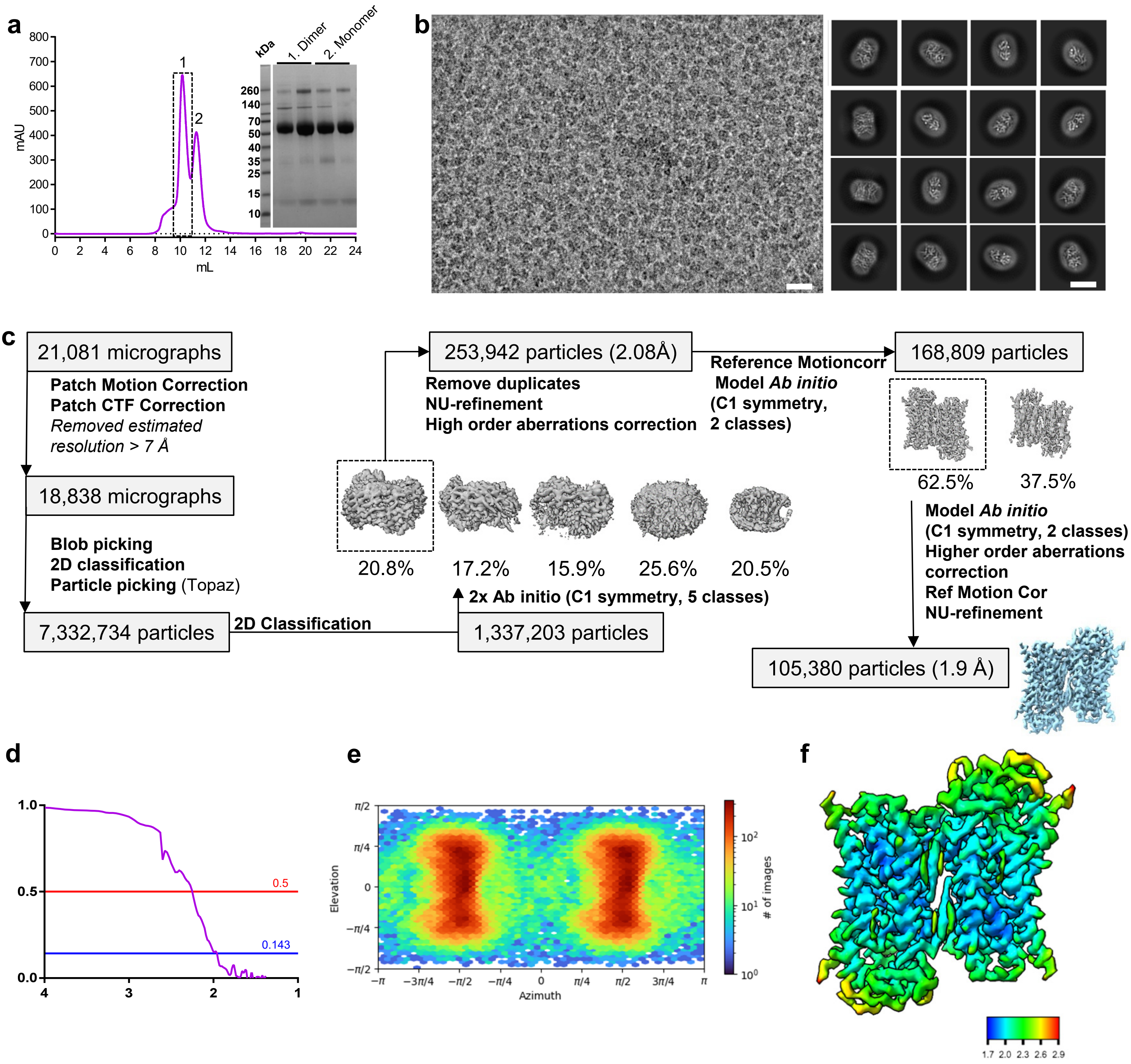


**Supplementary Figure 1. Biochemical characterization of $FZD_7$ and applied cryo-EM workflow.**
**(a)** Size exclusion chromatography (SEC) of purified $FZD_7$ on a Superdex 200 Increase 10/300 GL column with corresponding SDS-PAGE/Coomassie stain analysis of the peaks from the chromatogram taken from a single experiment. The dashed line shows the fraction taken for structural analysis. **(b)** Micrograph representation and gallery of class averages taken from 2D-classification from a single collection. **(c)** Cryo-EM workflow for data processing and refinement in cryoSPARC. See materials and methods for details. **(d)** The gold-standard Fourier shell correlation (FSC) curves for $FZD_7$ dimer representative of half-map with resolutions at 0.143 (blue) and 0.5 cutoffs (red). **(e)** The distribution of orientations over azimuth and elevation angles for particles used in the calculation of the final map. **(f)** Local resolution map generated by cryoSPARC of the reconstructed map.

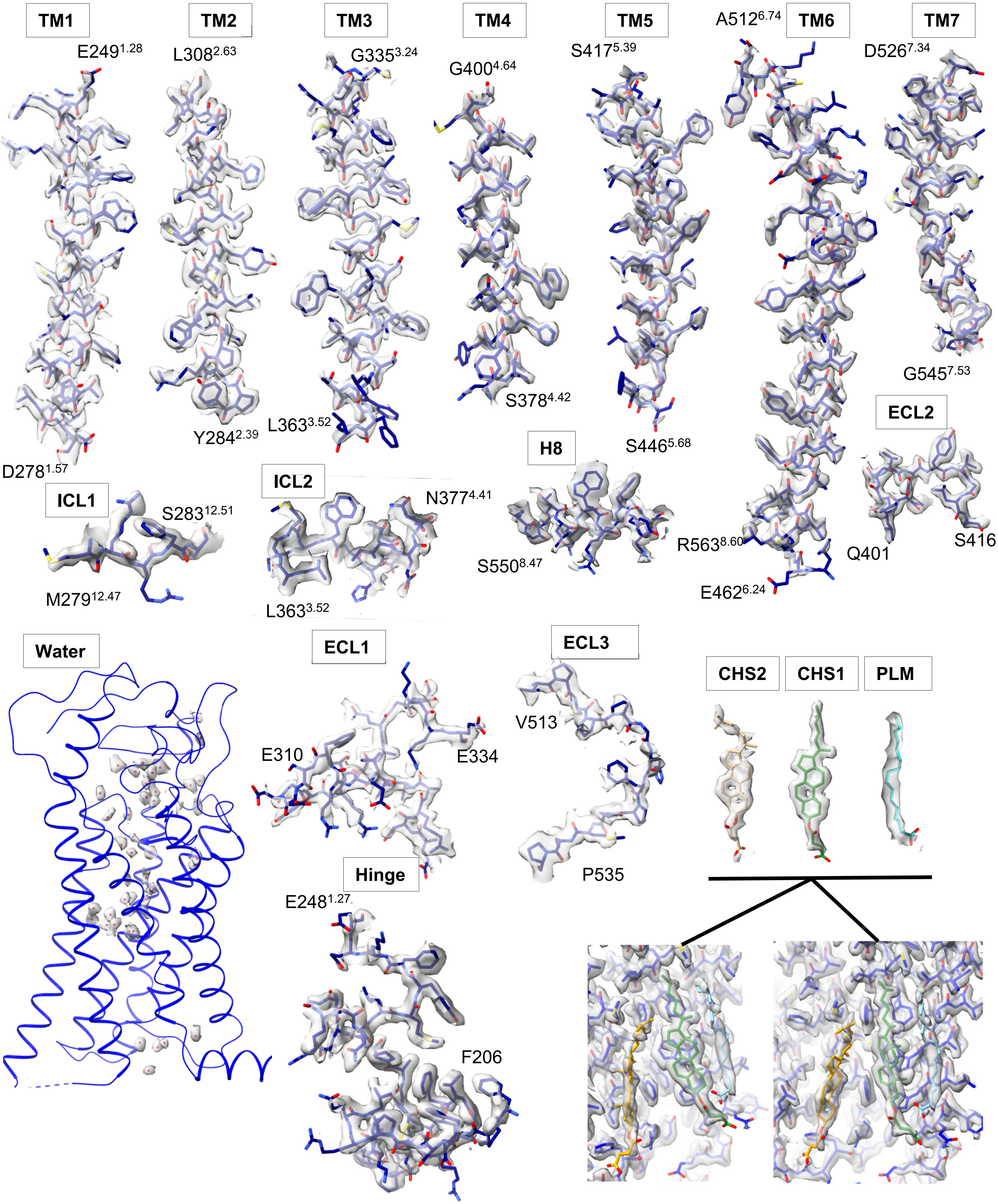


**Supplementary Figure 2. Cryo-EM maps with the model-fit of the inactive FZD$_7$ structure.** Representation of the cryo-EM map of the FZD$_7$ dimer shown in grey and contoured around each respective domain, region, or lipid, represented as sticks.

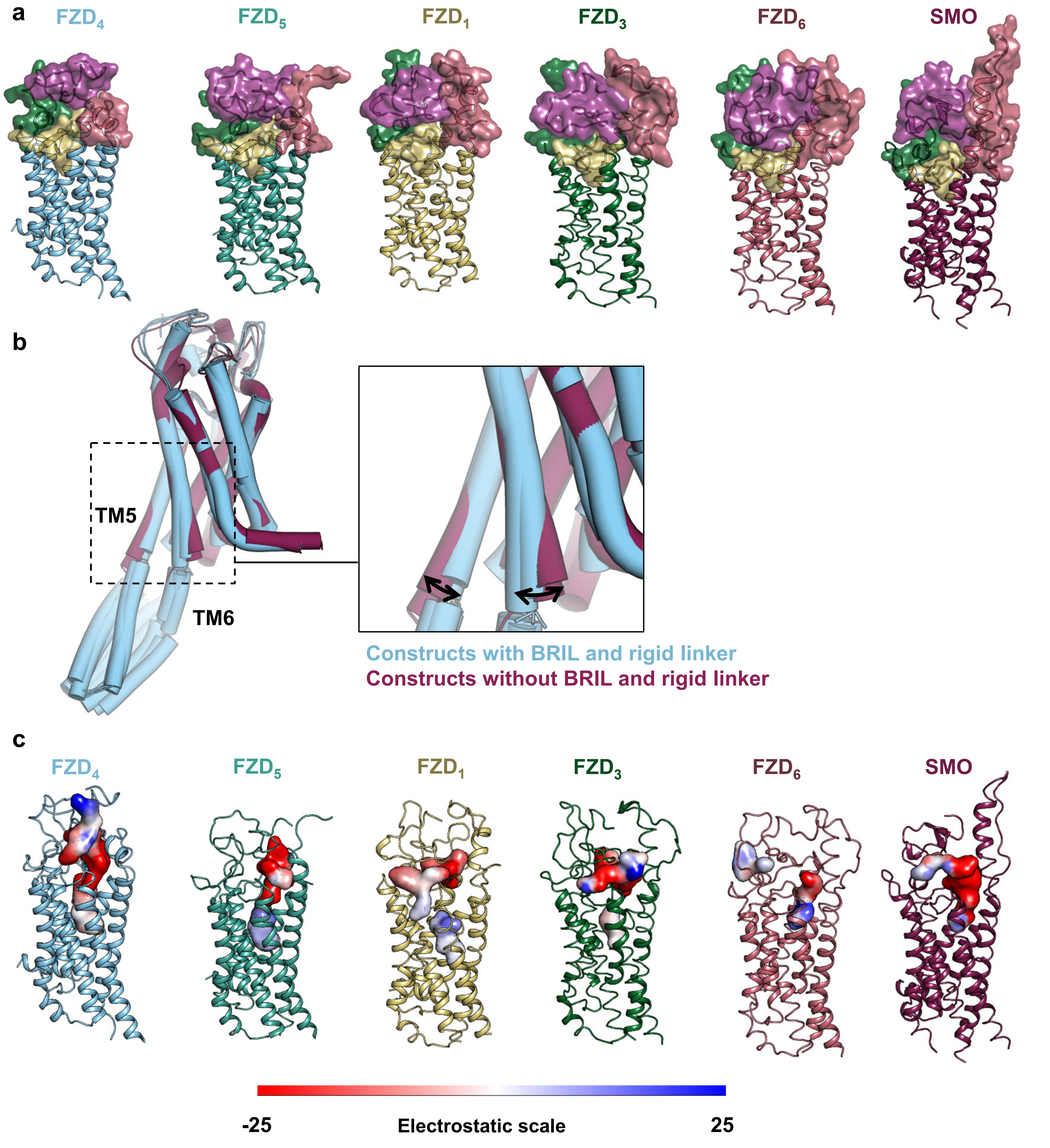


**Supplementary Figure 3. Organization of the peripheral lid, internal cavity, and TM6/7 dynamics of Class F GPCRs.** **(a)** Overview of representative FZDs and SMO in their respective homology clusters illustrating the distribution of the peripheral lid colored by ECL and hinge regions (ECL1; green, ECL2; yellow, ECL3; melon, Hinge; pink). **(b)** Superposition of inactive $FZD_7$ and $FZD_4$ (maroon) with BRIL-stabilized $FZD_{1,3,5,6}$ (blue) highlighting the dynamic nature of TM6 and TM7. **(c)** The internal cavity is depicted by the charge potential surface with negatively charged residues (red) and positively charged residues (blue). $FZD_4$ (PDB:6BD4) $FZD_5$ (PDB:6WW2) $FZD_1$ (PDB:8J9O) $FZD_3$ (PDB:8JHC) $FZD_6$ (PDB:8JH7) SMO (PDB:5L7D).

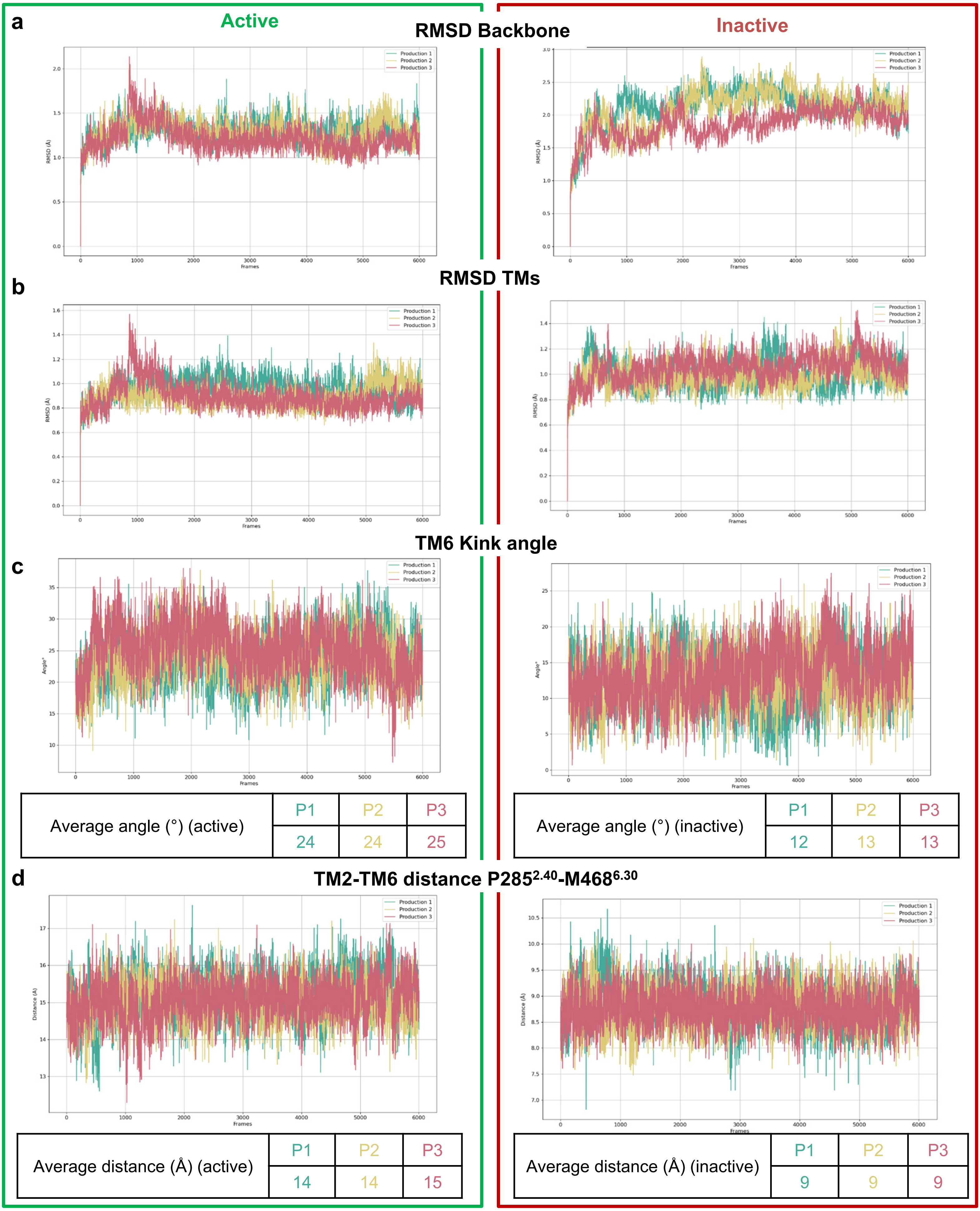


| Average angle (°) (active) | P1 | P2 | P3 |
|---|---|---|---|
| | 24 | 24 | 25 |

| Average angle (°) (inactive) | P1 | P2 | P3 |
|---|---|---|---|
| | 12 | 13 | 13 |

| Average distance (Å) (active) | P1 | P2 | P3 |
|---|---|---|---|
| | 14 | 14 | 15 |

| Average distance (Å) (inactive) | P1 | P2 | P3 |
|---|---|---|---|
| | 9 | 9 | 9 |

**Supplementary Figure 4. RMSD plots of $FZD_7$ backbone atoms and transmembrane domains with distance/angle calculations.**

RMSD of triplicate trajectories of the inactive and active conformations of $FZD_7$ depicting structural or conformational differences, in this case for **(a)** backbone atoms and **(b)** transmembrane helices. **(c)** The angular distance for replicates of the kink angle in TM6 and **(d)** the distance between residues at the base of TM2-TM6 comprising $P285^{2.40}$ and $M468^{6.30}$ is depicted over the course of the MD simulation for all replicates.

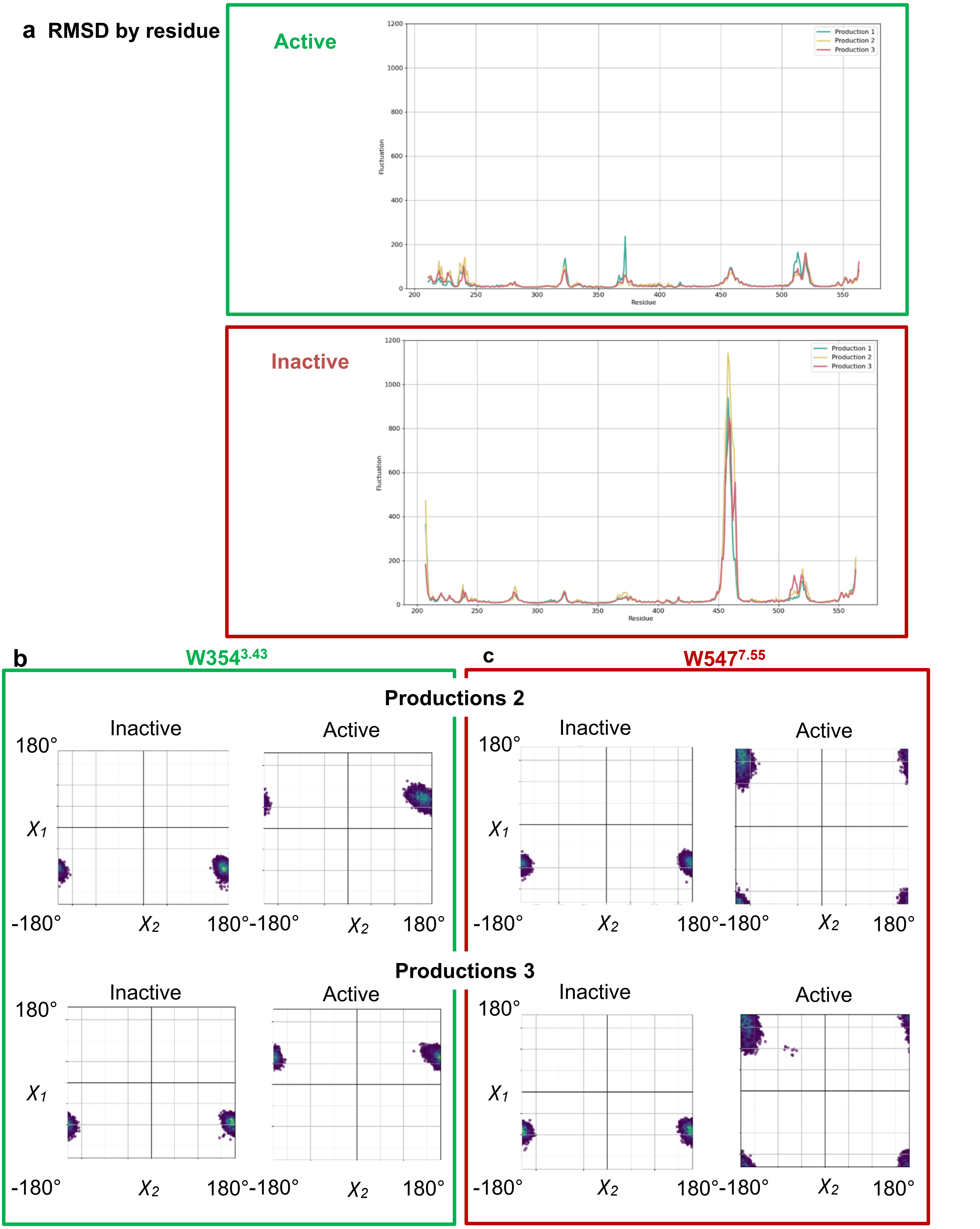


**Supplementary Figure 5. RMSD by residue plots of $FZD_7$ MDs and calculations and scatterplot of occurring χ1 χ2 dihedral angles.**
**(a)** RMSD by residue of triplicate trajectories of the inactive and active conformations. Scatterplot of occurring χ1 χ2 dihedral angles of residue **(b)** $W354^{3.43}$ and **(c)** $W547^{7.55}$ calculated for each frame (50 ps time steps) over the trajectory from replica 2 and 3 of $FZD_7$ in active and inactive state simulations.

**a**

| Residue | Interaction | Residue | Interaction |
|---|---|---|---|
| Q213$^{\mathrm{hinge}}$ | CO | G347$^{3.36}$ | CO |
| K215$^{\mathrm{hinge}}$ | CO | S350$^{3.39}$ | SC |
| Y222$^{\mathrm{hinge}}$ | SC | S351$^{3.40}$ | SC |
| R223$^{\mathrm{hinge}}$ | CO | G400$^{4.64}$ | CO |
| L225$^{\mathrm{hinge}}$ | NH | Q401$^{\mathrm{ECL2}}$ | SC, CO |
| A232$^{\mathrm{hinge}}$ | NH | V492$^{\mathrm{ECL2}}$ | CO |
| P233$^{\mathrm{hinge}}$ | CO | D403$^{\mathrm{ECL2}}$ | SC |
| L242$^{\mathrm{hinge}}$ | CO | G404$^{\mathrm{ECL2}}$ | CO |
| F245$^{1.24}$ | CO | D405$^{\mathrm{ECL2}}$ | SC |
| R254$^{1.33}$ | CO | L406$^{\mathrm{ECL2}}$ | NH |
| D278$^{1.57}$ | SC | S408$^{\mathrm{ECL2}}$ | CO |
| R280$^{\mathrm{ICL1}}$ | SC | Y412$^{\mathrm{ECL2}}$ | SC |
| S283$^{\mathrm{ICL1}}$ | SC, CO, NH | V413$^{\mathrm{ECL2}}$ | CO |
| E286$^{2.41}$ | SC, NH | G414$^{\mathrm{ECL2}}$ | NH |
| Y296$^{2.51}$ | SC | L429$^{5.51}$ | CO |
| H303$^{2.58}$ | SH, CO | R470$^{6.32}$ | SC |
| E317$^{\mathrm{ECL1}}$ | CO | Y478$^{6.40}$ | SC |
| Y324$^{\mathrm{ECL1}}$ | SC | A482$^{6.44}$ | CO |
| T326$^{\mathrm{ECL1}}$ | SC, NH | Y489$^{6.51}$ | SC |
| V327$^{\mathrm{ECL1}}$ | CO | E492$^{6.54}$ | SC |
| A328$^{\mathrm{ECL1}}$ | CO | R496$^{6.58}$ | SC |
| Q329$^{\mathrm{ECL1}}$ | SC, CO | S524$^{\mathrm{ECL3}}$ | SC |
| G330$^{\mathrm{ECL1}}$ | CO | P525$^{\mathrm{ECL3}}$ | CO |
| K332$^{\mathrm{ECL1}}$ | NH | K533$^{7.41}$ | SC |
| K333$^{\mathrm{ECL1}}$ | CO, NH | Y534$^{7.42}$ | SC |
| T337$^{3.26}$ | SC | M536$^{7.44}$ | CO |
| L343$^{3.32}$ | CO | T537$^{7.45}$ | SC, CO |

**b**

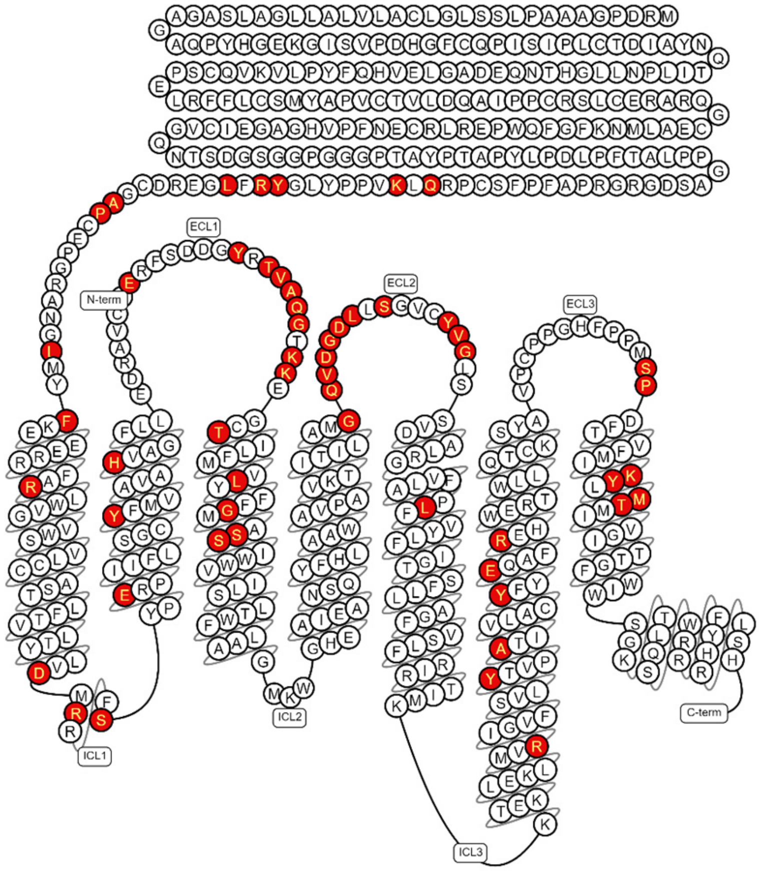


**Supplementary Figure 6. Residues involved in hydrogen bonds with water network.** **(a)** Table of residues involved in hydrogen bonding with the internal water network (SC: side chain; CO: $c_{\alpha}$ carbonyl NH: backbone amine) **(b)** $FZD_7$ snake plot representation with residues involved in hydrogen bonding with the internal water network highlighted in red.

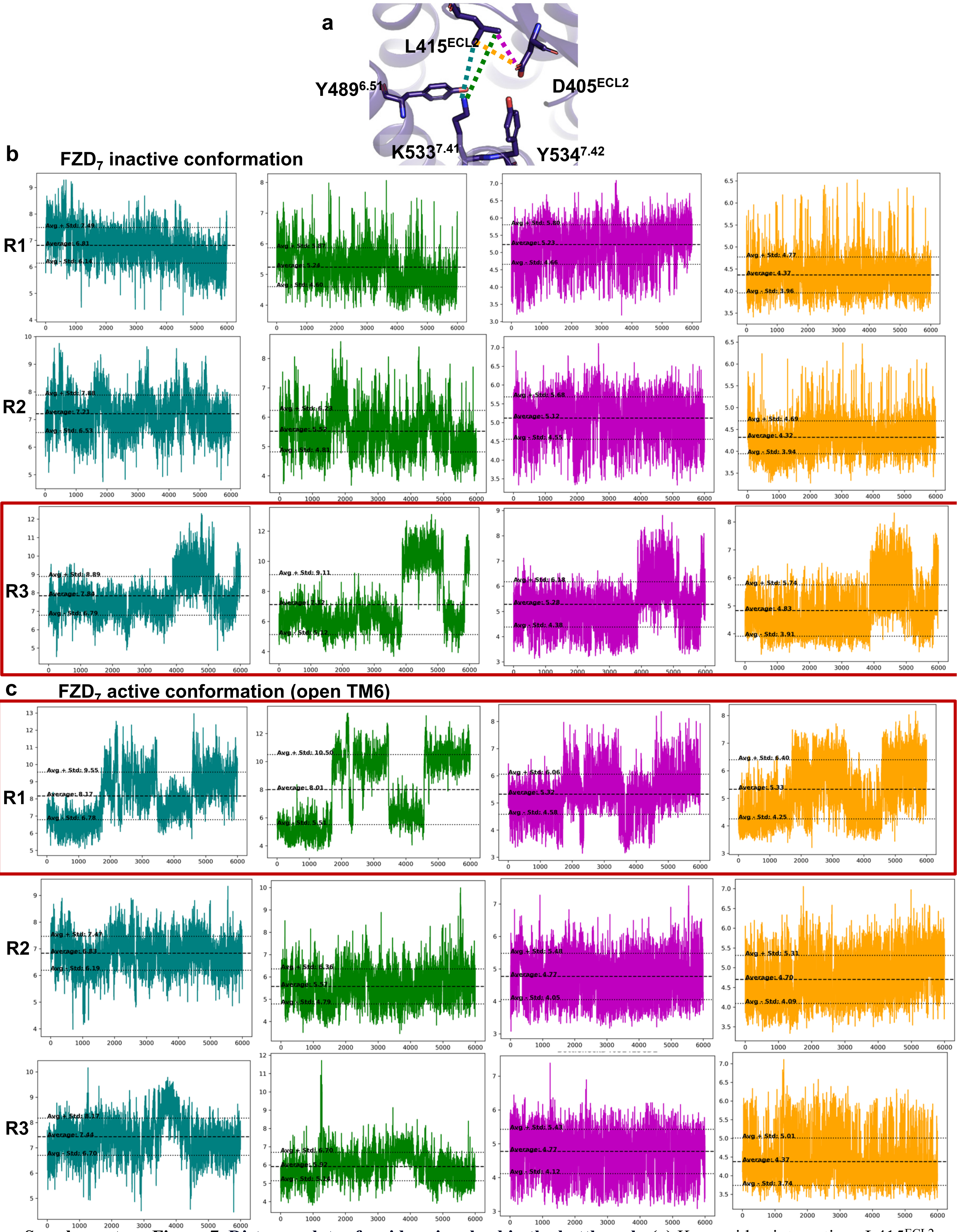

**Supplementary Figure 7. Distance plots of residues involved in the bottleneck. (a)** Key residue interactions L415$^{ECL2}$ Y489$^{6.51}$ (blue), L415$^{ECL2}$-K533$^{7.41}$ (green), D405$^{ECL2}$-L415$^{ECL2}$ (pink/yellow) involved in access of water molecules to the internal cavity, referred to as the bottleneck. The distances between the residues are measured over 300 ns using GROMACS for the **(b)** inactive and **(c)** active conformation depicted with averages (dotted line) ± standard deviation.

**a**

| CRD-hinge | TM1 | TM2-ECL1 | TM3 | TM4-ECL2 | TM5 | TM6 | TM7-H8 |
|---|---|---|---|---|---|---|---|
| C49 | F245$^{1.24}$ | Y284$^{2.39}$ | C336$^{3.25}$ | F381$^{4.45}$ | F424$^{5.46}$ | K463$^{6.25}$ | G541$^{7.49}$ |
| C57 | W256$^{1.35}$ | P285$^{2.40}$ | F340$^{3.29}$ | H382$^{4.46}$ | V425$^{5.47}$ | M468$^{6.30}$ | I542$^{7.50}$ |
| Y62 | T271$^{1.50}$ | F291$^{2.46}$ | Y344$^{3.33}$ | W386$^{4.50}$ | P4285.50 | R470$^{6.32}$ | W547$^{7.55}$ |
| T65 | T274$^{1.53}$ | C295$^{2.50}$ | F345$^{3.34}$ | P389$^{4.53}$ | G436$^{5.58}$ | G472$^{6.34}$ | I548$^{7.56}$ |
| Q76 | Y275$^{1.54}$ | Y296$^{2.51}$ | F346$^{3.35}$ | I394$^{4.58}$ | G443$^{5.65}$ | F474$^{6.36}$ | T553$^{8.50}$ |
| A79 | D278$^{1.57}$ | H303$^{2.58}$ | A349$^{3.38}$ | L397$^{4.61}$ | L447$^{5.69}$ | L477$^{6.39}$ | W557$^{8.54}$ |
| F86 | | C315 | W353$^{3.42}$ | V402 | I450$^{5.72}$ | C488$^{6.50}$ | |
| L89 | | | W354$^{3.43}$ | D405 | R451$^{5.73}$ | Y491$^{6.53}$ | |
| C94 | | | V355$^{3.44}$ | G409 | | E492$^{6.54}$ | |
| L102 | | | I356$^{3.45}$ | C411$^{45.50}$ | | W499$^{6.61}$ | |
| C103 | | | L357$^{3.46}$ | Y412$^{45.51}$ | | | |
| Y106 | | | W361$^{3.50}$ | V413$^{45.52}$ | | | |
| P108 | | | F362$^{3.51}$ | G414 | | | |
| C110 | | | | | | | |
| L123 | | | | | | | |
| C131 | | | | | | | |
| W142 | | | | | | | |
| P143 | | | | | | | |
| L146 | | | | | | | |
| C148 | | | | | | | |
| C210 | | | | | | | |
| C230 | | | | | | | |
| C234 | | | | | | | |

**b**

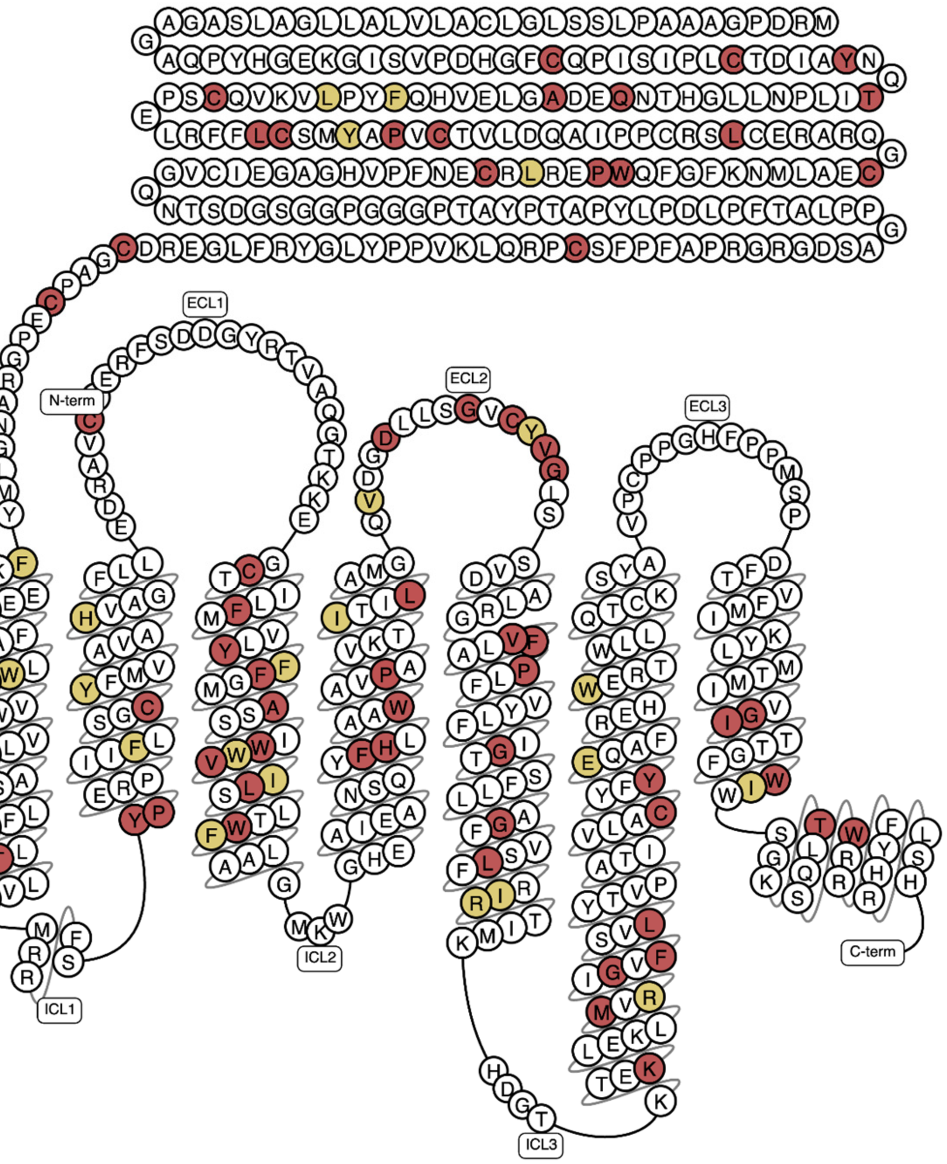


**Supplementary Figure 8. Conserved residues in Class F.**
**(a)** Table of conserved residues in Class F GPCRs. Fully conserved residues are colored in dark red and residues with conserved similarity are colored in yellow **(b)** FZD$_7$ snake plot representation of conserved residues in class F GPCRs with the same color code as in a).

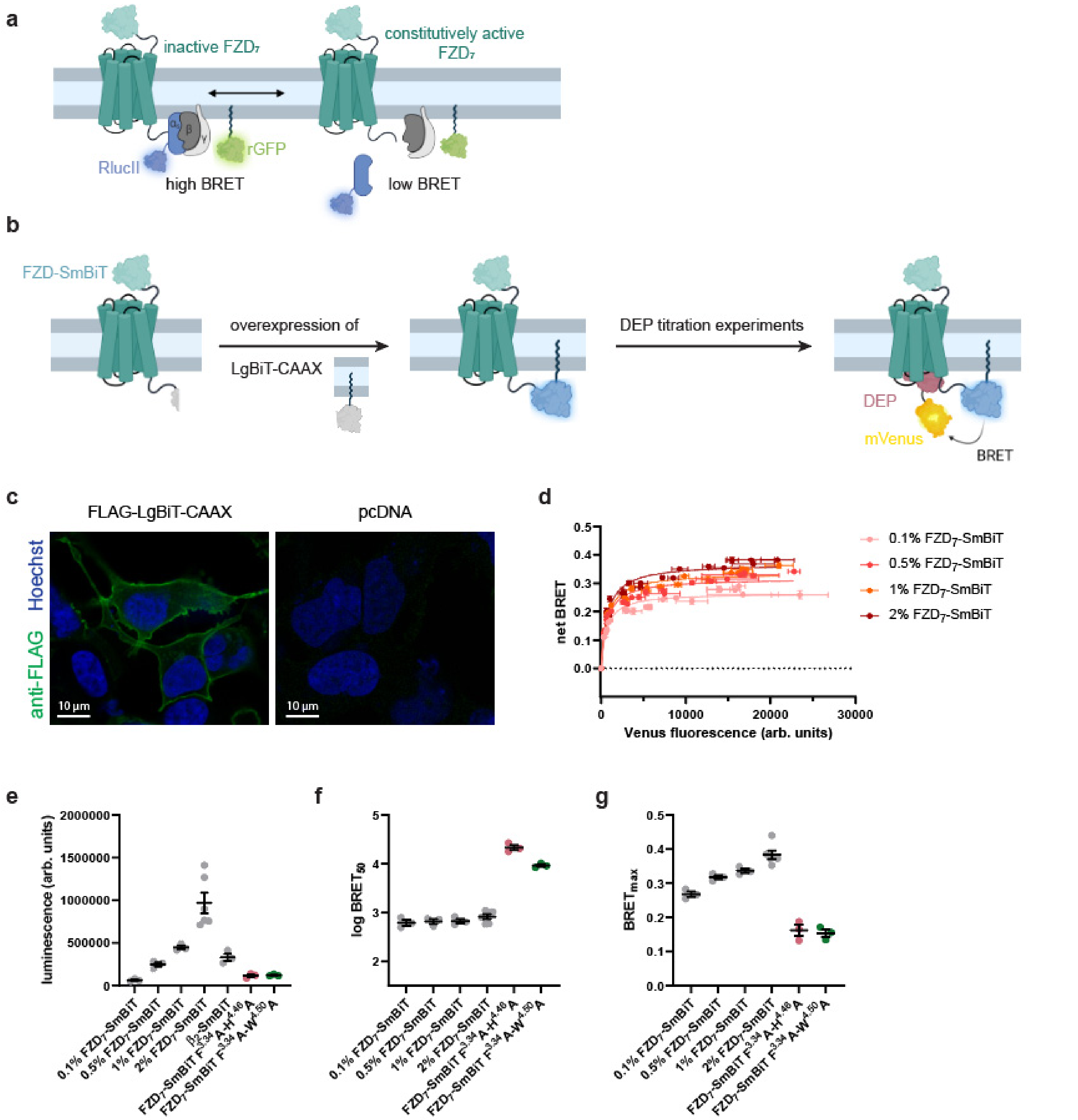


**Supplementary Figure 9. Functional validation setups employing the BRET Gαs translocation assay and SmBiT-LgBiT system.**

Schematic of the **(a)** BRET-based Gαs translocation assay to assess constitutive G protein coupling and **(b)** DEP recruitment assay using a split luciferase to detect BRET between receptor ($FZD_7$-SmBiT) and effector (DEP-mVenus). Assay schematics were created with BioRender. **(c)** Localization of FLAG-LgBiT-CAAX in the cell membrane in HEK293A cells by confocal light microscopy**.** Hoechst stain was used as nuclear counterstain. **(d)** Recruitment of DEP-mVenus to $FZD_7$-SmBiT. Experiments were performed in HEK293A cells transiently transfected with increasing amounts of plasmid encoding DEP-mVenus with multiple amounts of $FZD_7$-SmBiT-encoding plasmid. **(e)** Surface expression analysis of receptor-SmBiT constructs assessed via the split-luciferase system with membrane-tethered LgBiT-CAAX forming an active luciferase with $FZD_7$-SmBiT. **(f)** Log $BRET_{50}$ representing DEP-mVenus affinities with increasing amounts of $FZD_7$-SmBiT constructs and 1% of the indicated mutants. **(g)** $BRET_{max}$C values from DEP-Venus titrations with increasing amounts of $FZD_7$-SmBiT constructs and 1% of the indicated mutants. Cell surface expression plotted by **(h)** $logBRET_{50}$ and **(i)** $BRET_{max.}$ Data show mean ± SEM of five independent experiments performed in technical triplicates. Panel a,b were created with BioRender.com released under a Creative Commons Attribution-NonCommercial-NoDerivs 4.0 International license.

| | Inactive $FZD_7$ (EMD:EMD-19881 ) (PDB: 9EPO) | $FZD_7$-$G_s$ (PDB: 9EW2) |
|---|---|---|
| **Data collection and processing** | | |
| Magnification | 165,000 | |
| Voltage (kV) | 300 | |
| Electron exposure (e/A$^2$) | 80 | |
| Defocus range (μM) | -0.6 - 2.0 | |
| Pixel size (Å) | 0.5076 | |
| Symmetry imposed | C2 | |
| Initial particle images (no.) | 21,081 | |
| Map resolution (Å) | 1.93 | |
| FSC threshold | 0.143 | |
| Map local resolution range (Å) | 1.6 - 2.6 | |
| **Refinement** | | |
| Initial models used | 7EVW | 7EVW |
| Model resolution (Å) (FSC=0.5) | 2.1 | 3.45 |
| Map sharpening B factor (Å$^2$) | -45 | -100 |
| Model composition | | |
| Non-hydrogen atoms | 5860 | 8660 |
| Number of protein residues / atoms | 692 | 1090 |
| Number of ligands / ligand atoms | 6 | 0 |
| Average B factor (Å$^2$) | | |
| Protein | 81 | 72 |
| Ligands | 20 | |
| R.m.s deviations | | |
| Bond lengths (Å) | 0.005 | 0.005 |
| Bond angles (°) | 0.845 | 0.575 |
| Validation | | |
| Molprobity score | 0.7 | 0.96 |
| Clashscore | 0.73 | 2 |
| Poor rotamers (%) | 0.7 | 0.3 |
| Ramachandran plot | | |
| Favored (%) | 99.7 | 97.96 |
| Allowed (%) | 0.3 | 1.94 |
| Disallowed (%) | 0 | 0.1 |

**Supplementary Table 1. Cryo-EM data collection, refinement, and validation statistics.**
EMD; electron microscopy bank, PDB; protein data bank, RMSD; root mean square deviation.

| PDB ID | FZD/SMO | Resolution (Å) | State | MS contact (R/K$^{6.32}$ W$^{7.55}$) |
|---|---|---|---|---|
| 9EPO | $FZD_7$ | 1.9 | inactive | cation-π + Hbond backbone |
| 7EVW corrected | $FZD_7$ | 3.2 | active | cation-π + Hbond backbone |
| 8J9O | $FZD_1$ | 3.5 | inactive | cation-π |
| 8J9N | $FZD_1$ | 3.6 | active | cation-π + Hbond backbone |
| 8JH7 | FZD6 | 3.3 | inactive | cation-π |
| 8JHB | FZD6 | 3.4 | active | cation-π |
| 8JHC | FZD3 | 3.4 | inactive | cation-π |
| 8JHI | FZD3 | 3.5 | active | cation-π |
| 6BD4 | FZD4 | 2.4 | inactive | Hbond backbone |
| 6WW2 | FZD5 | 3.7 | inactive | cation-π |
| | | | | |
| 8CXO | SMO | 3.7 | inactive | Hbond backbone |
| 7ZI0 | SMO | 3.0 | inactive | cation-π + Hbond backbone |
| 6XBM | SMO | 3.2 | active | Hbond backbone |
| 6XBL | SMO | 3.9 | active | cation-π + Hbond backbone (R$^{6.32}$ T$^{7.54}$) |
| 6XBJ | SMO | 3.9 | active | Hbond backbone + Hbond backbone (R$^{6.32}$ W$^{7.57}$) |
| 6XBK | SMO | 3.2 | active | Hbond backbone + Hbond backbone (R$^{6.32}$ W$^{7.57}$) |
| 6O3C | SMO | 2.8 | active | Hbond backbone (R$^{6.32}$ W$^{7.57}$) + Hbond backbone (R$^{6.32}$ T$^{7.54}$) |
| 6OT0 | SMO | 3.9 | active | cation-π + Hbond backbone |
| 6D35 | SMO | 3.9 | active | cation-π |
| 6D32 | SMO | 3.8 | active | cation-π |
| 5V56 | SMO | 2.9 | inactive | cation-π + Hbond backbone (R$^{6.32}$ T$^{7.54}$) |
| 5V57 | SMO | 3.0 | inactive | cation-π + Hbond backbone + Hbond backbone (R$^{6.32}$ T$^{7.54}$) |
| 5L7I | SMO | 3.3 | inactive | cation-π + Hbond backbone + Hbond backbone (R6.32 T7.54) |
| 5L7D | SMO | 3.2 | inactive | cation-π + Hbond backbone + Hbond backbone (R$^{6.32}$ T7.54) |
| 4QIM | SMO | 2.6 | inactive | cation-π + Hbond backbone + Hbond backbone (R$^{6.32}$ T$^{7.54}$) |
| 4QIN | SMO | 2.6 | inactive | cation-π + Hbond backbone + Hbond backbone (R$^{6.32}$ T$^{7.54}$) |
| 4N4W | SMO | 2.8 | inactive | Hbond backbone + Hbond backbone (R$^{6.32}$ T$^{7.54}$) |
| 4JKV | SMO | 2.5 | inactive | cation-π + Hbond backbone |
| 4O9R | SMO | 3.2 | inactive | Hbond backbone (R$^{6.32}$ T$^{7.54}$) |

**Supplementary Table 2. Overview of molecular switch (R/K$^{6.32}$ -W$^{7.55}$) interactions in Class F structures.** A cutoff of 6Å distance as well as the position of the R guanidine group relative to aromatic position W indole group were considered for cation-pi interaction. A cutoff of 3.4Å distance was considered for hydrogen bonds. Hbond: hydrogen bonds. If not descsribed, Hbond occurs between R$^{6.32}$ side chain and W$^{7.54}$ backbone carbonyl.

| | Inactive $FZD_7$ ID: 2064 | Active $FZD_7$ ID: 2065 |
|---|---|---|
| **MD parameters** | | |
| Simulation box dimensions (nm) | 7.7 * 6.7 * 12.1 | 7.6 * 6.6 * 12.5 |
| total number of atoms | 64,511 | 65,888 |
| total number of water molecules | 10,000 | 10,000 |
| NaCl concentration (mM) | 150 | 150 |
| lipid composition | POPC (100%) | POPC (100%) |
| Protein | Chain A | Chain A |
| Ligand: Y01 (Cholesterol) | Chain B | Chain B |

**Supplementary Table 3. MD parameters table.**

| Primer name | Primer sequence (5' => 3') |
| --- | --- |
| muta XX-Nluc to XX-SmBiT_FW | GTTCGAGGAGATTCTCTAAGCGGCCGCGTTTAAA |
| muta XX-Nluc to XX-SmBiT_FW | AGCCGGTAGCCGGTCACAGAACCGCCACCTCCATC |
| #73_lin HA-FZD-XX_FW | GGCTCGAGTCTAGATGGAG |
| #74_lin HA-FZD-XX_RV | ATCCCGCATAATCCGGCAC |
| #345_$FZD_7$ for FZD7-XX_FW | CGTATGATGTGCCGGATTATGCGGGATCCCAGCCGTACCACG |
| #261_$FZD_7$ for FZD7-XX_RV | CCACCTCCATCTAGACTCGAGCCTACCGCAGTCTCCCCCTTG |
| #361_beta2 for HA-beta2-X_FW | TGATGTGCCGGATTATGCGGGATCCGGGCAACCCGGGAACG |
| #362_beta2 for HA-beta2-X_RV | CACCTCCATCTAGACTCGAGCCCAGCAGTGAGTCATTTGTAC |
| #283_lin DEPXXkRas_FW | CGAAAACATAAAGAAAAGATGAGCAAAG |
| #334_linDEPVkRas+FLAG_RV | GGATCCCTTGTCATCATCGTCCTTGTAGTCCATGGTGGCCAGAATTCGAAG |
| #332_LgBiT+FLAG overlap_FW | CAAGGACGATGATGACAAGGGATCCGTCTTCACACTCGAAGATTTCG |
| #333_LgBiT+kRas overlap_RV | GCTCATCTTTTCTTTATGTTTTCGACTGTTGATGGTTACTCGGAAC |
| muta $FZD_7$ F345A_FW | TTCATGGTGCTCTACGCCTTCGGCATGGCCAG |
| muta $FZD_7$ F345A_RV | CTGGCCATGCCGAAGGCGTAGAGCACCATGAA |
| muta $FZD_7$ H382A_FW | AACTCGCAGTACTTCGCCCTGGCCGCGTGGGC |
| muta $FZD_7$ H382A_RV | GCCCACGCGGCCAGGGCGAAGTACTGCGAGTT |
| muta $FZD_7$ W386A_FW | TTCCACCTGGCCGCGGCCGCCGTGCCCGCCGTC |
| muta $FZD_7$ W386A_RV | GACGGCGGGCACGGCGGCCGCGGCCAGGTGGAA |

**Supplementary Table 4. Primers Table.**
Primers used for design of mutants generated for functional assays**.**
