# Supplementary material for: Structural basis of frizzled 7 activation and allosteric regulation
Source: Nat Commun. 2024 Aug 28;15:7422. doi: 10.1038/s41467-024-51664-4 (PMC11358414; doi:10.1038/s41467-024-51664-4)
Supplement: Supplementary file 2 — Reporting Summary [file 41467_2024_51664_MOESM2_ESM.pdf]

nature portfolio

Corresponding author(s): Gunnar Schulte, Julien Bous

Last updated by author(s): Aug 2, 2024

Reporting Summary

Nature Portfolio wishes to improve the reproducibility of the work that we publish. This form provides structure for consistency and transparency in reporting. For further information on Nature Portfolio policies, see our Editorial Policies and the Editorial Policy Checklist.

Statistics

For all statistical analyses, confirm that the following items are present in the figure legend, table legend, main text, or Methods section.

n/a	Confirmed	
☐	☒	The exact sample size (*n*) for each experimental group/condition, given as a discrete number and unit of measurement
☐	☒	A statement on whether measurements were taken from distinct samples or whether the same sample was measured repeatedly
☐	☒	The statistical test(s) used AND whether they are one- or two-sided<br>*Only common tests should be described solely by name; describe more complex techniques in the Methods section.*
☒	☐	A description of all covariates tested
☐	☒	A description of any assumptions or corrections, such as tests of normality and adjustment for multiple comparisons
☐	☒	A full description of the statistical parameters including central tendency (e.g. means) or other basic estimates (e.g. regression coefficient) AND variation (e.g. standard deviation) or associated estimates of uncertainty (e.g. confidence intervals)
☐	☒	For null hypothesis testing, the test statistic (e.g. *F*, *t*, *r*) with confidence intervals, effect sizes, degrees of freedom and *P* value noted<br>*Give P values as exact values whenever suitable.*
☒	☐	For Bayesian analysis, information on the choice of priors and Markov chain Monte Carlo settings
☒	☐	For hierarchical and complex designs, identification of the appropriate level for tests and full reporting of outcomes
☒	☐	Estimates of effect sizes (e.g. Cohen's *d*, Pearson's *r*), indicating how they were calculated

Our web collection on statistics for biologists contains articles on many of the points above.

Software and code

Policy information about availability of computer code

Data collection	EPU software (v. 2.14.0) (Thermoscientific) was used for cryo-EM data acquisition<br>GROMACS(2023-2) was used to run the MD<br>Reader control software Tecan Spark plate reader<br>Excel office 365<br>CHARMM-GUI online server (https://charmm-gui.org/) for simulation system building<br>BLAST+ version 2.9.0 for search of similar sequences<br>MAFFT version 7.450 for alignment of similar sequences (paralogs and orthogs)<br>ClipKit version 1.1.3 for trimming multiple sequence alignments<br>FastTree version 2.1 for generation of the initial tree<br>IQ-TREE version 2.0.3 for determination of the model for maximum-likelihood tree generation<br>RAxML-NG version 1.0.3 for generation of orthologous<br>CD-HIT version 4.8.1 for identification of representative sequences used for alignment of human class F sequences
Data analysis	GraphPad Prism 10 (GraphPad Prism Software Inc.) was used to plot and analyses fluorescence based experiments<br>VMD (v1.9.4.a55) was used for MD analysis<br>plugin CavitOmiX (v. 1.1.beta, 2024, Innophore GmbH) in Pymol Molecular Graphics System (Schrödinger LLC) (v. 2.5) was used to analyses cavities<br>cryoSPARC (v4.2-v4.4) was use to process cryo-EM data<br>Topaz(v0.2.4) was used to pick the particles

Phenix (1.20.1-4487) ; Rosetta (2022.45+release.20a5bfe); Coot (0.9) were used for model refinment
Excel office 365
Amber tools 18 (cpptraj)
code and input files used for residue-residue contact analysis are provided at https://github.com/CompGenomeLab/fzd7_evolution_and_structure
DOI: 10.5281/zenodo.13175921

For manuscripts utilizing custom algorithms or software that are central to the research but not yet described in published literature, software must be made available to editors and reviewers. We strongly encourage code deposition in a community repository (e.g. GitHub). See the Nature Portfolio guidelines for submitting code & software for further information.

Data

Policy information about availability of data

All manuscripts must include a data availability statement. This statement should provide the following information, where applicable:

- Accession codes, unique identifiers, or web links for publicly available datasets
- A description of any restrictions on data availability
- For clinical datasets or third party data, please ensure that the statement adheres to our policy

Data and materials availability: The cryo-EM density maps for FZD7 dimer have been deposited in the Electron Microscopy Data Bank (EMDB) under accession codes EMD-19881. The coordinates for the models of the amended FZD7-Gs and for FZD7 dimer have been respectively deposited in the PDB under accession numbers 9EW2 and 9EPO. All molecular dynamic trajectories were deposited on GPCRmd. Multiple sequence alignments of paralogs and orthologs used for evolutionary analysis, and code and input files used for residue-residue contact analysis are provided at https://github.com/CompGenomeLab/fzd7_evolution_and_structure DOI: 10.5281/zenodo.13175921.

Research involving human participants, their data, or biological material

Policy information about studies with human participants or human data. See also policy information about sex, gender (identity/presentation), and sexual orientation and race, ethnicity and racism.

Reporting on sex and gender	N/A
Reporting on race, ethnicity, or other socially relevant groupings	N/A
Population characteristics	N/A
Recruitment	N/A
Ethics oversight	N/A

Note that full information on the approval of the study protocol must also be provided in the manuscript.

Field-specific reporting

Please select the one below that is the best fit for your research. If you are not sure, read the appropriate sections before making your selection.

- [x] Life sciences
- [ ] Behavioural & social sciences
- [ ] Ecological, evolutionary & environmental sciences

For a reference copy of the document with all sections, see nature.com/documents/nr-reporting-summary-flat.pdf

Life sciences study design

All studies must disclose on these points even when the disclosure is negative.

Sample size	No sample size calculation was performed. However, n > or = 3 is a typical sample size for biological experiments. Sample sizes including both number of independent experiments and technical replicates are stated in the respective figure legends.
Data exclusions	In general, no data were excluded. For some experiments, single wells were excluded from the analysis, if they were clearly not transfected. This could be estimated based on lack of mVenus fluorescence
Replication	At least three independent experiments were performed for each dataset found in the main text. The exact number of independent experiments and technical replicates is stated in the respective figure legends
Randomization	Randomization was not relevant for our study, as it was purely based on cell-based experiments assessing proteins interaction. These types of experiments do not necessarily require randomization.

Blinding	linding was not relevant for our study, as it was purely based on Cryo-EM and cell-based experiments. Analyzing these experiments is based on numerical data obtained from a transmission electron microscope and a plate reader and is therefore not subjective.

Reporting for specific materials, systems and methods

We require information from authors about some types of materials, experimental systems and methods used in many studies. Here, indicate whether each material, system or method listed is relevant to your study. If you are not sure if a list item applies to your research, read the appropriate section before selecting a response.

Materials & experimental systems

n/a	Involved in the study
☐	☒ Antibodies
☐	☒ Eukaryotic cell lines
☒	☐ Palaeontology and archaeology
☒	☐ Animals and other organisms
☒	☐ Clinical data
☒	☐ Dual use research of concern
☒	☐ Plants

Methods

n/a	Involved in the study
☒	☐ ChIP-seq
☒	☐ Flow cytometry
☒	☐ MRI-based neuroimaging

Antibodies

Antibodies used	Mouse Anti-Flag M2 antibody (Sigma #F1804) ,RRID: AB_262044<br>Alexa Fluor 488 conjugated goat anti mouse (invitrogen #A28175) , RRID: AB_2536161
Validation	Obtained for suppliers, no further validation

Eukaryotic cell lines

Policy information about cell lines and Sex and Gender in Research

Cell line source(s)	HEK293A , (Thermofisher scientific)/R70507<br>Delta FZD1-10 H293T cells , kind gift from Benoit Vanhollebeke (PMID:30026314)<br>Spodoptera frugiperda (Sf9) insect cells #11496015 (Thermo Fisher Scientific)
Authentication	No Further authentification
Mycoplasma contamination	All cell lines were tested negative to Mycoplasma contamination
Commonly misidentified lines (See ICLAC register)	none used

Plants

Seed stocks	N/A
Novel plant genotypes	N/A
Authentication	N/A
